# Nitric oxide in plants: an insight on redox activity and responses toward abiotic stress signaling

**DOI:** 10.1080/15592324.2023.2298053

**Published:** 2024-01-08

**Authors:** Khushboo Khator, Suman Parihar, Jan Jasik, Gyan Singh Shekhawat

**Affiliations:** a Plant Biotechnology and Molecular Biology Laboratory, Department of Botany (UGC-CAS) Jai Narain Vyas University, Jodhpur, India; b Institute of Botany, Plant Science and Biodiversity Centre, Slovak Academy of Sciences, Bratislava, Slovakia

**Keywords:** Nitric oxide, abiotic stress, antioxidant defense system, reactive oxygen species, protein modification and redox homeostasis

## Abstract

Plants, as sessile organisms, are subjected to diverse abiotic stresses, including salinity, desiccation, metal toxicity, thermal fluctuations, and hypoxia at different phases of plant growth. Plants can activate messenger molecules to initiate a signaling cascade of response toward environmental stresses that results in either cell death or plant acclimation. Nitric oxide (NO) is a small gaseous redox-active molecule that exhibits a plethora of physiological functions in growth, development, flowering, senescence, stomata closure and responses to environmental stresses. It can also facilitate alteration in protein function and reprogram the gene profiling by direct or indirect interaction with different target molecules. The bioactivity of NO can be manifested through different redox-based protein modifications including *S*-nitrosylation, protein nitration, and metal nitrosylation in plants. Although there has been considerable progress in the role of NO in regulating stress signaling, still the physiological mechanisms regarding the abiotic stress tolerance in plants remain unclear. This review summarizes recent advances in understanding the emerging knowledge regarding NO function in plant tolerance against abiotic stresses. The manuscript also highlighted the importance of NO as an abiotic stress modulator and developed a rational design for crop cultivation under a stress environment.

## Introduction

Globally, 90% of cultivated land is affected by various environmental stresses, including salinization, water deficit, extreme high and low temperatures, toxic metals, and herbicides. This percentage is increasing daily due to various natural and anthropogenic activities which are responsible for global climate change. These environmental stresses negatively influence plant growth and yield.^1^ Plants survive in stressful conditions by activating multiple stress adaptive responses.^1,2^ Programmed cell death and stress acclimation are major outcomes adopted by plants exposed to different stresses.^3^ Understanding the mechanisms of stress adaptation is a prerequisite for the development of crops with increased tolerance to various environmental fluctuations, which ultimately leads to increased crop production.^4,5,6^

In response to different environmental issues, the plant produces several redox molecules, namely reactive oxygen species (ROS) and reactive nitrogen species (RNS). However, the accumulation of these redox-active molecules is responsible for oxidative bursts in plant cells. Consequently, the regulation of cellular redox homeostasis is required for tolerance against abiotic stresses.^4^ Among redox molecules, nitric oxide (NO) is one of the dominant reactive nitrogen species which plays a dual function as an upstream and downstream regulator of the environmental stress response. The dual nature of NO mainly depends on its concentration and localization in the plant cell.^4,7^ Since the past decade, literature has reported the function of NO in abiotic stress tolerance, but our understanding physiological mechanisms of NO contribution to abiotic stress tolerance is still limited. Thus, the review mainly emphasizes the recent progress in NO function in abiotic stress response and the establishment of cellular redox homeostasis in plants.

## NO and its redox status

**Figure 1.**
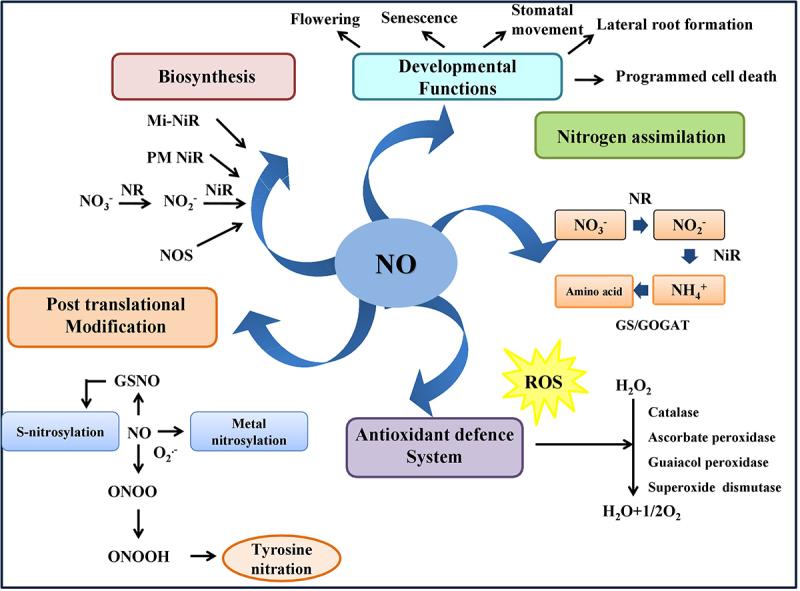
Multifunctional redox active molecule, i.e., NO, in plant cell. NO can regulates various developmental aspects including seed germination, flowering, lateral root development, stomatal closure, senescence and programmed cell death. It can also act as potent antioxidant which is involved in the regulatory mechanism of posttranslational modification and the nitrogen assimilation pathway.

Nitric oxide (NO) is an uncharged, gaseous free radical with a relatively short life (approx <6 s) in contrast to other free radicals. NO is a bioactive signaling molecule starting a journey as an environmental pollutant to “molecule of the year” in 1992. It exists in three redox-active forms, namely NO radical (NO.), nitrosonium cation (NO^+^) and nitroxyl anion (NO^−^).^8,9^ NO is soluble in H_2_O (0.047 cm^3^/cm^3^ H_2_O at 20°C, 1 atm), and the addition of ferrous salts increases its solubility.^10^ NO is a small diatomic molecule, which can easily migrate from both hydrophobic and hydrophilic regions of the cell. The outermost orbital of the NO molecule is occupied by an unpaired electron and as redox-active molecule, NO reacts with a broad range of targets such as metal complexes and other free radicals.^11^ Lipophilic NO can indirectly react with nucleic acids, lipids and proteins to produce multiple reactive derivatives such as peroxynitrite and *S*-nitrosothiols, collectively known as reactive nitrogen species.^12^ Moreover, NO rapidly reacts with other free radicals, such as superoxide anion (O_2_.^−^), to generate peroxynitrite (ONOO^−^), a potent oxidant. Additionally, the reaction between NO and glutathione (GSH) produces highly stable product *S*-nitrosoglutathione (GSNO), which is regarded as the dominant NO reservoir in plant cells.^9^ NO can also interact with an oxygen molecule to produce nitrogen dioxide (NO_2_). Moreover, NO_2_^−^ and NO_3_^−^areoxidized products synthesized after the oxidation of NO.^11^ NO at low concentrations can scavenge the hydroxyl radical (**∙**OH) that confers its antioxidant property (Figure 1).^13^ Besides the antioxidant properties of NO, it can also act as negative regulator of protein oxidation and lipid peroxidation.^14^ In addition, NO has been recognized as a multifunctional molecule that is involved in various developmental programs such as germination, flowering, lateral root formation, stomatal movement, senescence, programmed cell death and it is also implicated in nitrogen assimilatory pathway^2,4^ (Figure 1).

## NO homeostasis mediated by its synthesis and scavenging mechanisms

The cellular NO homeostasis in plants is necessary for this gaseous molecule to exhibit its signaling function. Thus, NO concentration needs to be balanced for various physiological and environmental stress conditions. The equilibrium between NO synthesis and scavenging mechanisms determines plants endogenous levels of this signal molecule.

## Synthesis of NO in plants

In Mammalia, NO is synthesized by three isoforms of NO synthase (NOS), neuronal (nNOS), endothelial (eNOS), and inducible (iNOS). NOS catalyzes the production of NO from L-arginine and molecular oxygen.^15,16^ Additionally, the NOS activity has been well documented in the algae *Ostreococcus tauri*.^17^While in the plants the production of NO from L-arginine by NOS was not proved,^4^ despite the pharmacological approach using NOS inhibitors suggests NOS-like activity in different plant species.^15^ However, in *Arabidopsis thaliana*, AtNOA1 (*Arabidopsis thaliana* NO-associated protein 1)/AtNOS, previously identified as a potential NOS, encodes GTPase, which is only indirectly involved in the accumulation of NO in response to abscisic acid (ABA).^18^

**Figure 2.**
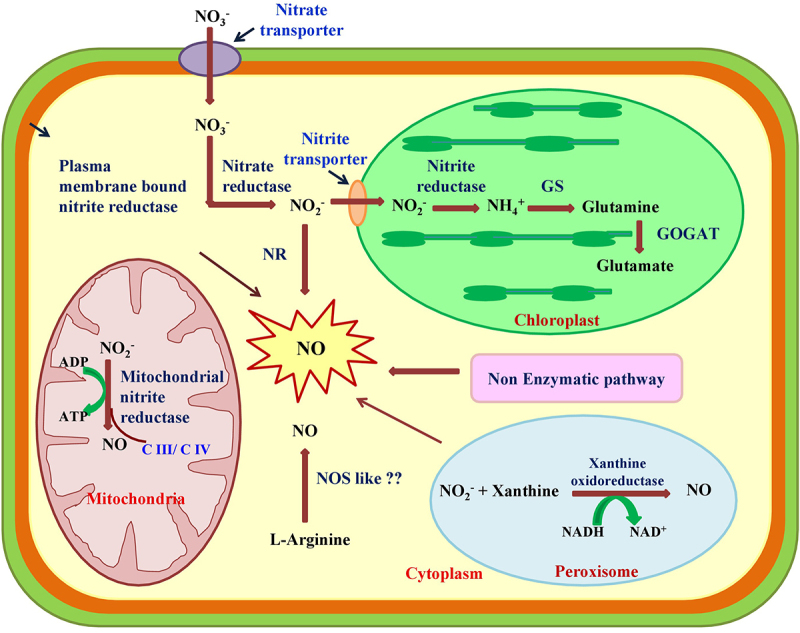
Overview of the biosynthesis of NO in different subcellular compartment of the plant cell. The production of NO from different enzymatic and nonenzymatic pathways includes NOS (nitric oxide synthase) like activity, NR (nitrate reductase), NiR (nitrite reductase) and XOR (xanthine oxidoreductase). L- arginine dependent NOS like activity exist in plant but their function and biochemical nature needs further investigation. NR catalyzes NADPH dependent reduction of nitrate to nitrite which is further reduced to NO. The XOR catalyzes conversion of nitrite to NO by using the NADH or xanthine as a reducing substrate.

Genetic and pharmacological approaches indicate that nitrate reductase (NR) is another NO biosynthetic enzyme in plants. It catalyzes NADPH-dependent reduction of nitrate to nitrite in plants (Figure 2); however, *in vivo* and *in vitro* analysis revealed that NR may also catalyze the reduction of nitrite to NO and its derivative peroxynitrite (ONOO^−^).^4,19^ Potassium cyanide, sodium azide, and tungstate are inhibitors of NR which inhibits the production of NO in higher plants.^20,21^ The production of NO via NR is affected by various factors like concentration of nitrate and nitrite, cellular pH, posttranslational modification, etc.^22,23^

In the roots of *Nicotiana tabacum*, plasma membrane-bound NR was found to be coupled with a nitrite: NO oxidoreductase (Ni: NOR) (Figure 2). The activity of a couple of enzymes was only detected in roots, not in leaves, which suggests its function as an indicator of nitrate availability in the soil.^4^

Another reductive route for NO synthesis is the mitochondrial electron transport chain.^15,22^ In mitochondria, NO production occurs at very low oxygen concentration, estimated at approximately 20 µM. The enzyme mitochondrial nitrite reductase (NiR) is prominent producer of NO in diverse organisms including algae, yeast, mammals and plants. Nitrite is used as a substrate for mitochondrial NiR and acts as a terminal electron acceptor in the electron transport system in some yeast. Plant mitochondria are also involved in the reduction of nitrite by enzyme complex of ETC, i.e., cytochrome oxidase (C III) and cytochrome reductase (C IV).^22,24^

The peroxisomal enzyme xanthine oxidoreductase (XOR) catalyzes the reduction of nitrite to NO. In higher plants, xanthine oxidoreductase (XOR) exists in two inter-convertible forms, i.e., xanthine oxidase and xanthine dehydrogenase.^25^ Predominantly, xanthine oxidase produces superoxide and uric acid aerobically, whereas under anaerobic condition, XOR from bovine milk is involved in the reduction of nitrite to NO, and NADH and xanthine are used as reducing substrates.^26^ In addition, XOR plays a significant role in NO production of in *Lupinus albus* roots under phosphate deficiency.^27^ Finally, NO is produced by various oxidative routes namely via polyamines and hydroxylamine.^15^ In *Arabidopsis thaliana*, spermine and spermidine are important polyamines, which trigger NO production.^28^ In the hydroxylamine-mediated NO pathway, superoxide directly interacts with hydroxylamine to produce NO (Figure 2).^29^

In addition to the enzymatic pathway, NO can be synthesized by non-enzymatic mechanisms.^15^ At acidic pH, nitrite is chemically reduced to generate NO.^30^ This was documented, e.g., for *Hordeum vulgare* and *Brassica juncea*,^31,32^ In chloroplasts and apoplastic space, ascorbic acid reduces nitrite to form NO and dehydroascorbic acid. Carotenoids are involved in the light-regulated reduction of NO_2_^−^, another possible mechanism for NO nonenzymatic production.^10,31^

## NO scavenging mechanism in plants

Besides synthesis, the scavenging mechanisms are responsible for maintaining NO intracellular level. These processes are regulated by several specific enzymes. NO can react with the reduced form of glutathione (GSH) to produce *S*-nitrosoglutathione (GSNO), which is the major reservoir of NO and its efficient donor for protein *S*-nitrosylation. In plant cells, the level of *S*-nitrosoglutathione (GSNO) is regulated by *S*- nitrosoglutathione reductase 1 (GSNOR 1).^33^. This enzyme is mainly localized in the cytosol and produces *S*-nitrosoglutathione (GSNO), an oxidized form of glutathione (GSSG) and ammonia (NH_3_) by the catalytic reduction of S-nitrosoglutathione (GSNO) using NADH as the reducing equivalent. GSNO and NO have been documented to be involved in the development and responses to stress environments.^34^ The transcriptomic analysis of GSNOR null mutants revealed that maximum GSNOR expression was documented in roots and leaves tissue and upregulating genes involved in iron and redox homeostasis, whereas downregulating genes increased the resistance against pathogens. This finding confers that GSNOR deficient plant alters cellular redox homeostasis and further provides evidence that the GSNOR-regulating enzymes are actively involved in plant defense against pathogens.^35^ Besides these, GSNOR deficient plant also shows altered thermo tolerance, declined plant growth, enhanced inflorescence number and deformity in the branching of stem and trichomes.^34,35^

However, NO reacts with molecular oxygen to generate nitrite in an aerobic environment. Nitrite and nitrate can also be produced by an aqueous solution of NO. NO can be scavenged by interacting with reactive oxygen species. NO can also interact with superoxide to produce the nitrating agent peroxynitrite (ONOO-).^15^ The redox reaction between NO and other reactive molecules is as follows.

$$\begin{array}{c} 2NO\cdot +\;O_{2}⟶2NO_{2}\cdot \\ 2NO\cdot +\;O_{2}⟶N_{2}O_{4}\;\;\overset{H_{2}O}{⟶}\;\;NO_{2}\cdot +\;NO_{3}-\\ 2NO\cdot +\;O_{2}\cdot -⟶OONO^{-}\overset{H^{+}}{⟶}NO_{3}\cdot +\;\;H^{+}\\ NO^{+}+H_{2}O_{2}⟶OONO-+2H^{+} \end{array}$$

The interaction of NO with reactive oxygen species and lipid peroxyl radical (LOO·) generates nitro fatty acids.^36^. Besides this, the cellular homeostasis of NO can be controlled by its oxidization to NO_3_^−^, which is facilitated by truncated and non-symbiotic haemoglobins. Haemoglobins are reduced to Fe (II) hemoglobin, which is further reduced to dioxygenate NO. In *Arabidopsis thaliana* and *Solanum tuberosum* mitochondria, the scavenging mechanism of NO is mainly O_2_ dependent process, and the mechanism is also involved in the regulation of NO inhibition during respiration.^37^ Additionally, the non-symbiotic hemoglobin is also involved in the oxidative degradation of NO to produce nitrate, which is another important mechanism for the consumption of NO during hypoxic conditions. Thus, the expression of hemoglobin and NO homeostasis is co-ordinately controlled by nitrate.^38^

## *S*-nitrosothiol regulated NO homeostasis via the nitrogen assimilation pathway

It is well documented that the reduction of NO_2_^−^ by nitrite reductase produces ammonium which is further incorporated into amino acids. Moreover, NO_2_^−^ is used as a substrate for NO production, indicating a direct correlation between the nitrogen assimilation pathway and the NO signaling cascade. The recent study by Frungillo et al..^39^ demonstrated that NO plays an important role in the nitrogen assimilation pathway by regulating the uptake and reduction of nitrate. The *Arabidopsis* mutant plant with defective NO homeostasis revealed that increased *S*- nitrosothiol (SNO) reduces the affinity of nitrate transporters which leads to a decrease in the nitrate uptake by roots. Noticeably, the activity of NR is also negatively influenced by the level of SNO. Thus, a negative correlation occurs between SNO and nitrate uptake or reduction and consequently, the production of NO or SNO is reduced. Hence, the nitrate content and amino acid homeostasis in plants are mainly regulated by *S*- nitrosothiol.^39^

Furthermore, the study by Frungillo et al.^39^ has demonstrated that NO also regulates its scavenging mechanisms in plants.^39^ High nitrate accumulation inhibits the activity of *S*- nitrosoglutathione reductase, which is associated with NR-regulated NO formation. By post-translational modification, NO can *S*- nitrosylate GSNOR, thus inhibiting its activity and preventing the degradation of GSNO. These results suggest that NO regulates its homeostasis through GSNO, which is a major NO reservoir that regulates feedback inhibition of the nitrate assimilation pathway. The study further confers that NO acts as a sensor of nitrogen availability and potentially regulates plant growth and development.^39^

## NO: mode of action in plants

### Posttranslational modifications

**Figure 3.**
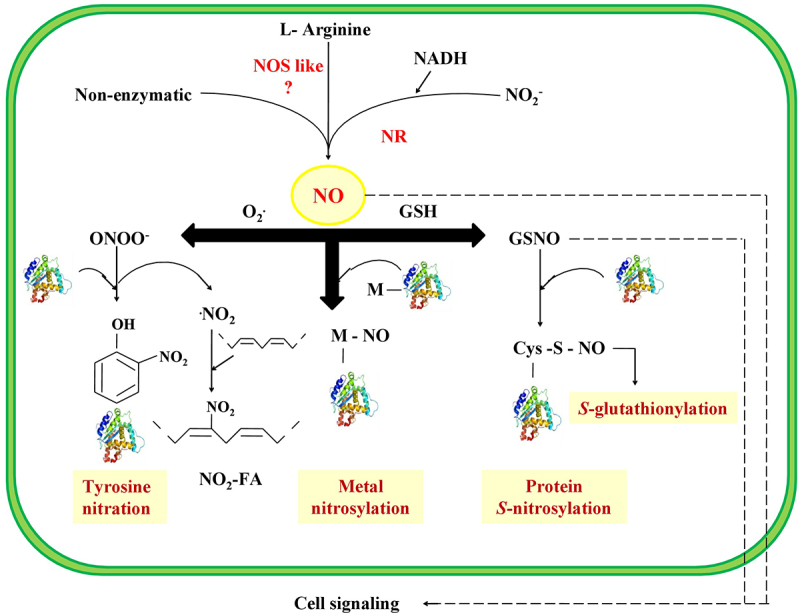
The proposed model of NO regulated post translational modification in plants. L-arginine dependent NOS like enzyme produces nitric oxide which can interact with reduced glutathione in the presence of oxygen to produced *S*- nitrosoglutathione (GSNO). The reaction between NO and superoxide radical produces peroxynitrite which can effects the protein nitration process. Nitric oxide can react with transition metals to produce metal-nitrosyl complexes. *S-*glutathionylation is cysteine based post translational modification comprises of disulfide bridge between cys protein and glutathione.

NO can react with transition metals such as iron (Fe^3+^), copper (Cu^2+^), or zinc (Zn^2+^) to form metal-nitrosyl complexes (M-NO).^40^ Metal peroxidation is inhibited by metal nitrosylation, thereby preventing the production of reactive oxygen species.^13^ NO can interact with iron in heme groups, which is the most relevant interaction in biological systems, and NO is more efficiently bound with Fe^2+^ than Fe^3+41^ Furthermore, the peroxynitrite (OONO^−^), a reactive derivative of NO, may cause tyrosine nitration of protein by adding a nitro group to the tyrosine side chain.^42^ Among the various post-translation modifications, *S*-nitrosylation is one of the most crucial protein modifications mediated by NO. *S*-nitrosothiol is produced by the attachment of NO to the – SH (thiol) group of cysteine (Cys) residues (Figure 3).

*S*-nitrosylation illustrates a dynamic mechanism for the regulatory function of protein in animal systems and emerges as a redox-based protein modification required for the survival of plants .^43^ *S*-nitrosothiol is formed by the covalent bonding between the thiol groups, and NO and this interaction can be labile due to its redox and light sensitivity (Figure 3). *S*- nitrosylation can regulate various protein activities. In recent studies, protein *S*-nitrosylation has been recognized as the key mechanism in response to different abiotic stresses.^44^ For example, carboxylase activity of ribulose-1,5-bisphosphate carboxylase/oxygenase was documented to be inhibited by *S*- nitrosylation of a large subunit of Rubisco enzyme in *B. juncea* exposed to cold stress.^45^ Conversely, the activity of cytosolic ascorbate peroxidase was enhanced by *S*- nitrosylation in *Pisum sativum*.^46^ On the other hand, phytochelatin (PC2, PC3, and PC4), the cysteine-rich metal binding peptide, has not been influenced by *S*- nitrosylation in Cd-treated Arabidopsis plants.^47^ Wang et al.^48^ conducted a study on *Boehmeria nivea* leave reported that NO might regulate Cd-induced cytotoxicity by the *S*- nitrosylation of antioxidant enzymes. In this context, the level of *S*- nitrosylation and activity of antioxidants was decreased by the reduced production of NO under Cd-induced stress. Conversely, the application of SNP triggers the *S*- nitrosylation level and activity of antioxidants, signifying that the redox-based protein modification, i.e., *S*-nitrosylation potentially involved in the NO-mediated mitigation of Cd toxicity.^48^ Further, glutathione (GSH) tripeptide antioxidant can directly replace SNOs and produces *S*- nitrosoglutathione (GSNO), the most stable pool of NO. Consequently, the dynamic equilibrium is present between the level of *S*-nitrosoglutathione (GSNO) and total cellular protein *S*- nitrosothiol (SNO) in plants. In addition, *S*-nitrosoglutathione reductase (GSNOR) is the most decisive enzyme regulating the total cellular *S*-nitrosylation level by reducing GSNO.^4,49^

Tyrosine nitration is another posttranslational modification directly affecting different functions of protein, including alteration in enzyme activity, proteolytic degradation, and phosphorylation level (Figure 3). In plants, various environmental stresses elicited the nitration of tyrosine residues which is recognized as an indicator of nitrosative stress.^12^ Several reports document that the nitration of proteins is influenced qualitatively and quantitatively by different environmental constraints. For example, in *Helianthus annuus* infected by pathogen *Plasmopora halstedii*, the increased *S*-nitrosothiol level is accompanied by an augmented rate of tyrosine nitrated polypeptide. The nitration of tyrosine in proteins was increased in the leaves and suspension culture of *Nicotiana tabacum*.^50^ A recent study revealed that both the protein modification, namely nitration of tyrosine residue and *S*- nitrosothiol, simultaneously regulated the function of cytosolic enzyme ascorbate peroxidase.^46^ Moreover, in *Arabidopsis thaliana*, the reorganization of microtubules was triggered by protein nitration under the nitrosative stress, which may be the most relevant mechanism stimulating plant growth and development.^51^

NO also triggers the alteration in gene expression by regulating different transcription factors and mitogen-activated protein kinases.^12,52,53^ It is well established that NO may directly regulate the expression of many genes, which are involved in various physiological processes such as metabolism, photosynthesis, production and detoxification of ROS, defense against abiotic stress, signal transduction, disease resistance and cellular trafficking.^54^ The transcriptomic analysis conducted by Polaveri et al.^55^ in *A. thaliana* treated with the NO donor sodium nitroprusside reveals that from 2500 transcripts, 120 of them altered levels. Furthermore, in the microarray analysis of Parani et al.^56^, representing approximately 24,000 genes, 342 were shown to be up regulated and 80 down regulated by NO. About 10% of the NO-regulated genes encoded different transcription factors including members of the ethylene response factor (ERF) family, Myb-related transcription factors, WRKY-type transcription factors and zinc finger proteins. These transcription factors are implicated in diverse biological functions in plants, including signal transduction, defense mechanisms, cellular detoxification, and biosynthesis of ethylene, jasmonic acid, lignin, and alkaloid. WRKY and MYB transcription factors and some other genes are regulated by GSNO. In conclusion, NO may interact with the complex networks of metabolites and also regulate differentially expressed genes in plants.^52^

## NO in responses to abiotic stress

**Figure 4.**
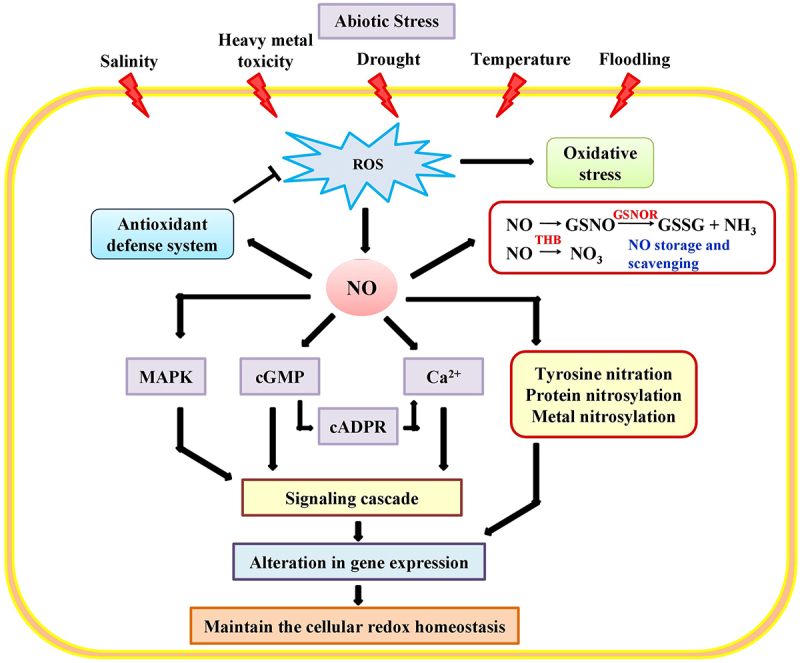
Regulatory role of NO in plants response to abiotic stress tolerance. NO interacts with different target molecules such as mitogen-activated protein kinases (MAPKs), cyclic guanosine monophosphate (cGMP), cyclic adenosine diphosphoribose (cADPR) and calcium (Ca^2+)^ to counteract the ROS mediated oxidative burst in plant. As the protection against oxidative stress, NO can initiate the cascade of signaling events which altered the expression of gene and also facilitates the reestablishment of cellular redox homeostasis.

The dual mode of NO action NO is manifested by its cytotoxic and cytoprotective properties in responses to various abiotic stresses. NO production is enhanced in different plants under various environmental stresses. NO at lower concentrations regulates ROS-mediated cytotoxicity. NO may also eliminate NO_2_, which is toxic for plants at high levels. Furthermore, it is well documented that NO participates in jasmonic acid biosynthesis, reactive oxygen species (H_2_O_2,_ O_2_., OH^.,1^O_2_) scavenging mechanisms, and regulation of the expression of stress-responsive genes (Figure 4).^57^

## Salt stress

Soil salinization is one of the major threats to agricultural crop production resulting from large-scale water irrigation. Due to increasing daily food demand, maximum land area is cultivated artificially at a greater level, which leads to over-accumulation of soluble salts in the plant soil.^58–61^ Generally, sodium chloride and sodium sulfate are the most frequent soluble salts in the soil. Besides this, the soil also contains substantial amounts of sodium bicarbonate (NaHCO_3_), potassium nitrate (KNO_3_), calcium sulfate (CaSO_4_), and in water partially dissolving magnesium sulfate (MgSO_4_). However, soil salinity is mainly due to two principal ions, i.e., Na^+^ and Cl^−^. Physiologically, soil salinity is regarded as a top limiting factor for plant productivity and development due to the multiple adverse effects, including ionic imbalance, osmotic stress, and oxidative burst in plants (Figure 4).^58,62^

The involvement of NO in salinity stress tolerance mechanisms has been documented in different plant species (Table 1). Earlier research suggests that the exogenous application of sodium nitroprusside as a NO donor can defend the plant from salt-induced oxidative damage, maintain cellular ionic homeostasis and improve plant growth.^81^ *Arabidopsis thaliana Atnoa1*plants with impaired NOS activity were shown to be more vulnerable to salt and oxidative stress.^82^ Treatment of mutant plants with sodium nitroprusside attenuated the NaCl-induced Na^+^ to K^+^ ratio.

**Table 1.** Nitric oxide involved in the regulation of antioxidant defense metabolism and ROS detoxification in different plant species exposed to salinity stress.

Plant Species	Tissue exposed	Concentration and duration of NaCl	Concentration and duration of SNP	NO mediated effects on plant	Reference
*Aegiceras corniculatum*	Leaves	300 mM NaCl, 30 days	100 µM SNP, 30 days	Decreased the oxidative stress by reducing the MDA and ROS content and increased the activity of antioxidant enzyme	^63^
*Brassica juncea*	Leaves	100 mM NaCl, 20 days	100 µM SNP, 20 days	Reduced the MDA and ROS content and improved the activity of antioxidants	^64^
*Brassica juncea*	Leaves	100 mM NaCl, 35 days	100 µM SNP, 15 days	Decreased the ion leakage and MDA content and enhanced the proline accumulation	^65^
*Cicer arietinum*	Leaves	50 mM and 100 mM NaCl, 45 days	50 µM SNAP, 45 days	Enhanced the plant growth, proline content and ROS scavenging activity	^66^
*Cucumis sativus*	Leaves	50 mM NaCl, 8 days	100 µM SNP, 8 days	Activation of antioxidant defense system and reduced the oxidative stress	^67^
*Glycine max*	Shoots, roots and nodules	80 mM NaCl, 16 days	10 µM DETA, 16 days	Decreased the accumulation of ROS and increased the ascorbate eroxidise	^68^
*Jatropha curcas*	Endosperm and embryo axis	100 mM NaCl, 4 days	75 µM SNP, pretreatment of seeds	Increased the plant growth, decreased the electrolyte leakage and accumulation of toxic ions	^69^
*Kosteletzkya virginica*	Leaves and roots	200–400 mM NaCl, 5 days	60 Mm SNP, 5 days	Reduced the oxidative damage by the stimulation of ROS scavengers	^70^
*Lupinus luteus*	Roots	200 mM NaCl, 48 hrs	100 µM SNP, 48 hrs	Enhanced the root growth and induced the cellular defense	^71^
*Lycopersicon esculentum*	Seedlings	100 mM NaCl, 8 days	100 Mm SNP, 8 days	Decreased the membrane integrity and induced the activity of ROS quenchers	^72^
*Pisum sativum*	radicals	100 mM and 400 mM NaCl, 7 days	100 µM SNP, 7 days	Reduced the malonaldehyde level and improved the antioxidant activity	^73^
*Oryza sativa*	Seedlings	0–100 mM NaCl, 8 days	Less than 1 µM SNP, pretreatment for 2 days	Enhanced antioxidant activity mitigates the salt induced oxidative stress in rice seedlings	^74^
*Helianthus annuus*	Seedlings	120 mM NaCl, 10 days	100, 250, 500, 1000 µM SNP, 10 days	Decreased ROS accumulation and induced the level of antioxidants	^75^
*Solanum lycopersicon*	Leaves and roots	120 mM NaCl, 8 days	100 and 300 µM SNP, 8 days	Higher accumulation of proline and ascorbate and reduced the electrolyte leakage	^76^
*Triticum aestivum*	Seedlings	100 mM NaCl, 15 days	100 µM SNP, 15 days	Improved the plant biomass by enhancing the photosynthetic rate and upregulation of antioxidants	^77^
*Triticum aestivum*	Seedlings	150 mM NaCl, 65 days	100 µM SNP, pretreatment of seeds for 12 hrs	Enhanced the total phenolic content and decreased the oxidative burst	^78^
*Triticum aestivum*	Seedlings	100 mM NaCl, 30 days	50 µM SNP, 30 days	Improved the photosynthetic efficiency and reduced the oxidative stress	^79^
*Avicennia marina*	Leaves	400 mM NaCl, 30 days	100 µM SNP, 30 days	Improved chl and photosynthesis rate	^80^
*Brassica juncea*	Seedlings	10 mM-100 mM NaCl, 96 hrs	10–200 µM SNP, 96 hrs	Enhanced proline accumulation and upregulation of antioxidants	^59^

Similarly, in salt-treated *Populus euphratica*, NO increases K^+^/Na^+^ ratio via H_2_O_2,_ and this process mainly depends on the enhanced plasma membrane H^+^-ATPase activity. The application of NO has been found to induce the plasma membrane H^+^-ATPase expression and enhance the salt tolerance by increasing the K^+^ /Na^+^ ratio in the callus of *Phragmites communis*.^83^ as well. It was speculated that the NO pre-treatment maintained the Na^+^ and K^+^ ion homeostasis and increased starch degradation to produce total soluble sugars.^84^ The study by Wu et al.^85^ on *Solanum melongena* has shown that photosynthetic capacity was improved by protecting the photosynthetic pigments after NO treatment. Furthermore, exogenously applied NO also enhances the photosynthetic efficiency in *B. juncea* plants exposed to NaCl stress.^86^ In addition, NO regulates stomatal conductance and increases the RuBisCo enzyme’s activity, leading to increased photosynthesis under NaCl stress.^86^ In plant cells, osmotic adjustment is mainly associated with the stomatal conductance.

NO is recognized as a potential inhibitor of reactive oxygen species causing peroxidation of lipids and oxidation of proteins.^4^ NO may trigger the tolerance mechanisms toward NaCl by inducing oxidative stress in different plant species. For instance, pre-exposure to NO leads to the upregulation of antioxidant enzyme activity including superoxide dismutase, catalase, and ascorbate peroxidase, minimizes the membrane’s permeability, accumulation of reactive oxygen species and MDA level in *Cucumis sativus*.^67^ Subsequently, in NaCl-treated *Cicer arietinum*, 0.2 mM sodium nitroprusside raises the activity of ascorbate peroxidase (APX) and guaiacol peroxidase (GPX).^87^ The study by Tanou et al.^88^ on citrus leaves stated that the application of NO and hydrogen peroxide elicited the long-lasting activity of antioxidants under NaCl-induced oxidative stress.

NO is recognized as a potential inhibitor of reactive oxygen species that cause peroxidation of lipids and oxidation of proteins.^4^ NO has played a vital role in plant defense as a redox signaling molecule. Interaction of NO with other defense molecules such melatonin reduced the levels of reactive oxygen species, free toxic radicals, lipid peroxidation and improved the antioxidant enzyme activity during sodic alkaline toxicity. NO has been demonstrated to modulate plant adaptation to different stresses through interaction with melatonin. Yan et al.^89^ reported that the application of NO with melatonin induces nitrate reductase activity to synthesized NO and maintained K+/Na+ balance in rice by regulating H+ pump activity of tonoplast and plasma membrane under salinity stress. NO-melatonin interaction induced NaCl tolerance mechanism in tomato plant.^90^

NO may trigger the tolerance mechanisms toward NaCl by inducing oxidative stress in different plant species. For instance, pre-exposure to NO leads to the upregulation of antioxidant enzyme activity including superoxide dismutase, catalase, and ascorbate peroxidase, minimizes the membrane’s permeability, accumulation of reactive oxygen species and MDA in *Cucumis sativus*.^67^ Subsequently, in NaCl-treated *Cicer arietinum*, 0.2 mM sodium nitroprusside raises the activity of ascorbate peroxidase (APX) and guaiacol peroxidase (GPX).^87^ The study by Tanou et al.^88^ on citrus leaves stated that the application of NO and hydrogen peroxide elicited the long-lasting activity of antioxidants under NaCl-induced oxidative stress.

Moreover, NO has been played considerable role in cell protection against the NaCl induced stress due to the upregulation of antioxidant machinery in different plant species, such as *Kosteletzkya virginica*.^70^, *Triticum aestivum* L.^91^, and *Cicer arietinum* L.^92^

Hasanuzzaman et al. ^93^ conducted a study on *T. aestivum* under salt stress (300 mM NaCl) in combination with 1 mM SNP and observed that the presence of NO enhances the activity of both enzymatic and non-enzymatic antioxidants. Similarly, reports on most of the plant species such as tomato.^72^, mangrove.^63^, Indian mustard.^65^ and pea.^73^ show that NO exerts the positive effects on antioxidant defense machinery and reactive oxygen species metabolism. Strikingly, 2,2′(hydroxyl nitroso hydrazono) bis-ethanimine (DETA) and *S*-nitroso-N-acetyl penicillamine (SNAP) are another NO-producing compounds to counteract the NaCl-induced stress by up regulating the antioxidant enzyme activity and reducing MDA and ROS level.^66^ Additionally, the study carried out by Ali et al.^78^ and Gadelha et al.^69^ suggested that seeds treated with 0.1 mM and 75 µM sodium nitroprusside, respectively, and showed positive results against 150 and 100 mM NaCl, respectively. Consequently, NO plays a regulatory role in the alleviation of salt-induced oxidative stress to a certain extent.

However, NO-mediated oxidative and nitrosative signaling and its associated protein modifications including carbonylation of protein, nitration of tyrosine residue, and *S*-nitrosylation orchestrate that the citrus plants acclimated to NaCl stress.^94^ In response to salinity stress, NO regulates different kinds of protein kinases which is proposed as an essential component of the signaling cascade. In tobacco BY-2 cells exposed to salinity stress, NO triggers the activation of NtOSAK (*Nicotiana tabacum* Osmotic Stress-Activated Protein Kinase). It was well established that a glycolytic enzyme glyceraldehyde-3-phosphate dehydrogenase (GAPDH) was found to interact with NtOSAK. The short-time exposure of NaCl stimulated the *S*- nitrosylation of GAPDH which leads to no alteration in its *in vivo* activity. Additionally, the phosphorylation of NtOSAK and its interaction with GADPH are not affected by the *S*- nitrosylation of GAPDH. Thus, NO may directly or indirectly regulate the activity of both proteins, i.e., GAPDH and NtOSAK.^95^ In cell suspension of *A. thaliana*, the proteomic analysis reveals that ascorbate peroxidase is one of the potential targets for *S*-nitrosylation.^96^ Further, short-term and long-term exposure to NaCl were found to be reduced the extent of protein *S*-nitrosylation.^97,98^ Collectively, the documented results suggest that NO plays a vital role in minimizing the salt induced stress. The antioxidant defense system, redox modification of ROS quenchers, and photo respiratory pathways are various underpinning mechanisms that might be regulated by NO.

## Drought stress

**Figure 5.**
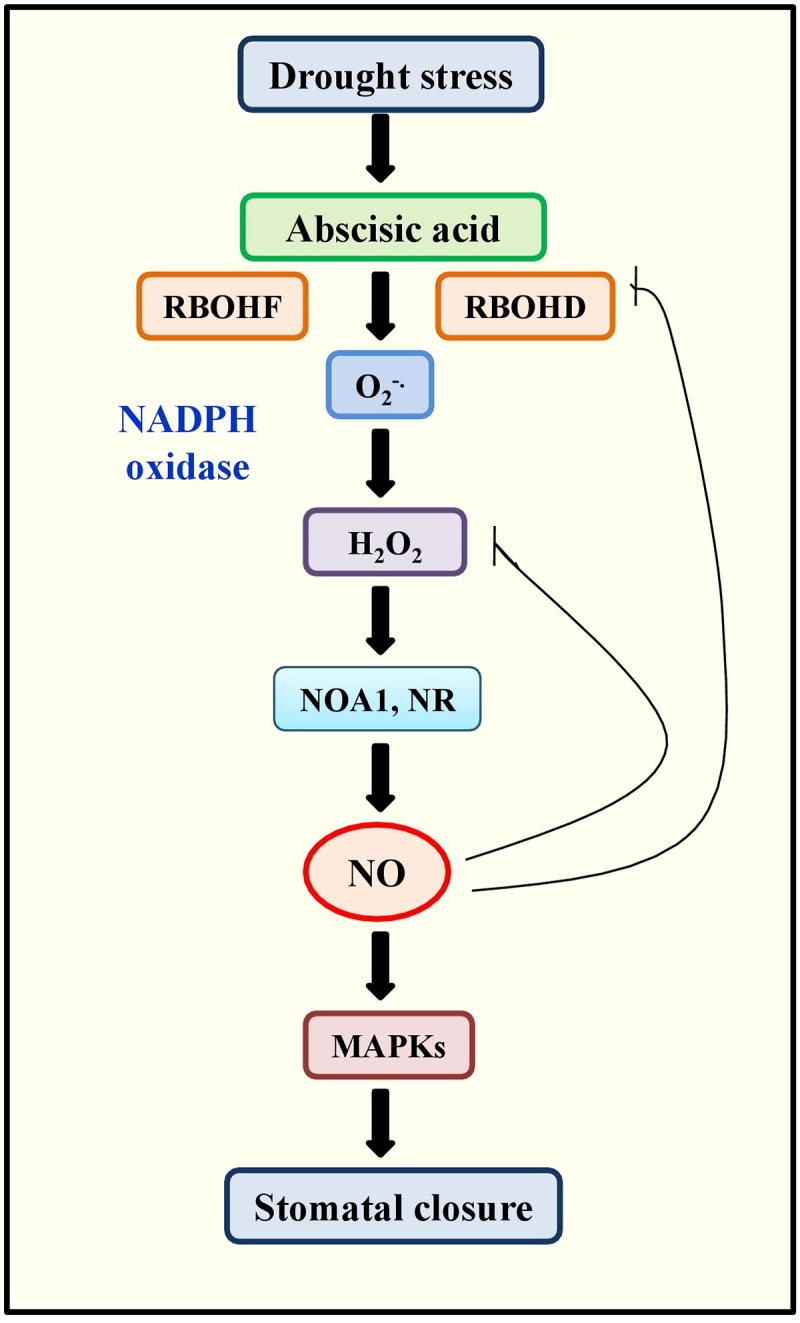
Schematic model showing the regulation of NO in the stomatal closure during the drought stress. In dessicated environment, the accumulation of ABA was induced by the activation of NADPH oxidases and RBOHD and RBOHF(Respiratory burst oxidase homolog D and F) which can triggers the increased level of H_2_O_2_ leading to the NO production. NO can activate MAPK signaling cascade which results in closing of stomata.

Water-deficit is the major devastating factor for crop yield because it impairs physiological processes such as the uptake of nutrients and photosynthesis.^7^ The ameliorating effect of NO in preventing drought stress has been reported for different plant species.^99^ The genetic and pharmacological studies suggest that NO is essential for ABA induced fractional closure of stomata, resulting the increased plant drought tolerance.^100^ In guard cells of *A. thaliana*, the stomatal closure mainly depends on the ABA-induced generation of NO. Consistently, the double *nia1 nia2* mutant of NR with reduced NO production did not show ABA-induced stomatal closure.^101^ Generally, ABA is accumulated in plant during drought stress, and superoxide is produced by the activation of RBOHD and RBOHF (respiratory burst oxidase homolog D and F) NADPH oxidase enzymes. This redox signaling produces NR-mediated NO and further stimulates MAPK (mitogen activated protein kinase) signaling, which facilitates the closing of stomata (Figure 5).^20,101^

During water deficit, NO can improve the accumulation of ABA, which can be inhibited by using the scavenger of NO, i.e., cPTIO [2-(4-Carboxyphenyl)-4, 4, 5, 5 tetramethylimidazoline-1-oxyl-3-oxide].^102^ ABA signaling may be regulated by OST1/SnRK2.6 kinase, which is S-nitrosylated in response to ABA. Its kinase activity is inhibited by the S-nitrosylation at Cys137.^103^ In addition, GSNO has also attenuated the kinase’s inhibitory activity by a Cys137 to Ser (C137S) mutation.

In *T. aestivum*, applying SNP increased the relative water content and mitigated the drought-induced oxidative stress, thereby enhancing the plant growth. In cucumber plants, drought resistance is enhanced by polyamines and cytokinins-mediated NO production.^104^ The activities of antioxidants, namely SOD and CAT, were found to be increased with enhanced ABA levels and improved tolerance toward drought stress in Bermuda grass cultivars.^105^ Filippou et al.^106^ reported that excessive NO concentrations inhibited NR activity, which might be responsible for mitigating the nitrosative stress in drought-stressed seedlings of *Ailanthus altissima.*

In *Antiaris toxicaria, desiccation induces over-accumulation of* ROS which blocks recalcitrant seed germination.^107^ In this context, the application of NO efficiently decreases desiccation-induced ROS accumulation and improved seed germination by upregulation of antioxidant defense system. Furthermore, analysis of the posttranslational modifications reveals that pre-treatment with NO inhibited the carbonylation of different antioxidant enzymes such as ascorbate peroxidase (APX), dehydroascorbate reductase (DHAR), glutathione *S*-transferase (GST), glutathione reductase (GR) and peroxidase (POX). Additionally, NO can trigger another protein modification, *S*-nitrosylation of APX, DHAR and GR can be triggered by NO.

Finally, NO may act through transcription factors. S-nitrosylation of AtMYB30 and AtMYB2, which participates in both stress conditions salinity and drought, causes the reduction of their DNA binding capacity.^108^ In conclusion, the presented data show the vital role of NO in drought tolerance by regulating various redox switches and transcription factors and protein kinase modifications.

## Heavy metal stress

Plants are exposed to the surrounding microenvironment, including metals. Metals exhibit a positive and negative effect on the plant, dependent on type and source (Figure 4). NO emerged as a critical modulator in tolerance mechanism against toxic metals, including Cd.^109,110^, Cu.^111^, Ni.^112^, Zn.^113^, and As.^114^ The contribution of NO to responses to metal toxicity in different plant species is summarized in Table 2. The ways in which NO participates in the tolerance to metals are diverse. In the *A. thaliana* peroxisomes, lead-induced excessive NO accumulation increased the activity of antioxidant enzymes.^133^ NO also positively effects on primary and lateral root development in plants exposed to excess metal concentrations.^134^ In *Lupinus luteus*, the application of a NO donor, SNP reduces the superoxide level in their roots under metal stress.^71^ Similarly, the pretreatment with SNP mitigates Cd-induced stress in sunflowers and other crops.^135,136^ In *Medicago trunculata*, NO was suggested to enhance Cd tolerance by the proline and glutathione accumulation.^137^

**Table 2.** Role of NO in different plant species exposed to heavy metal induced oxidative stress.

Plant species	Metal compounds	Plant organs exposed	Metal conc.	NO Conc.	NO regulated response in plants	References
*Citrus grandis*	AlCl_3_	Seedlings	1200 µM	10 µM	Reduced the oxidative stress by promoting the plant growth and root metabolism	^115^
*Triticum aestivum*	AlCl_3_	Seedlings	30 µM	250 µM	Enhanced the activity of antioxidants	^116^
*Oryza sativa*	Na_2_HAsO_4_	Seedlings	100 µM	100 µM	Improved the photosynthetic pigments, plant growth and changed the expression analysis of silicon transporter	^117^
*Oryza sativa*	NaAsO_2_	Roots/coleoptile	25 µM and 50 µM	50 µM	Altered the architecture of roots and improved the accumulation of antioxidants	^118^
*Pistia stratiotes*	Na_2_HAsO_4_	Plants	1.5 mg/L	0.1 mg/L	Induced the expression of antioxidants and inhibit the negative effect on the photosynthetic pigments	^119^
*Vigna radiata*	Na_2_HAsO_4_	Sprouting seeds	100 µM and 250 µM	50 µM	Reduced the inhibition of seed growth due to As and increased the accumulation of antioxidant enzymes	^120^
*Vicia faba*	Na_2_HAsO_4_	Plants	100 µM, 200 µM and 400 µM	50 µM	Enhanced the plant growth, chlorophyll contents and level of plant growth harmone	^121^
*Triticum aestivum*	Na_2_HAsO_4_	Seedlings	250 µM and 500 µM	250 µM	Improved the leaf water content, level of osmolyte and chlorophyll content and increased the activities of antioxidant enzymes	^122^
*Arachis hypogaea*	CdCl_2_	Leaves	50 µM, 100 µM and 200 µM	250 µM	Increased photosynthetic efficiency and level of ROS quencher	^123^
*Brassica juncea*	CdCl_2_	Plants	5 µM −200 µM	10 µM −2000 µM	Enhanced the activities of antioxidants and improved the leaf water content and chlorophyll content	^124^
*Brassica juncea*	CdCl_2_	Seedlings	50 µM and 100 µM	100 µM	Improved chlorophyll content and Enhanced the activities of antioxidants	^2^
*Cucumis sativus*	CdCl_2_	Plants	100 µM	100 µM	Inhibition of leaf chlorosis and strengthened the ROS scavenging activity	^125^
*Festuca arundacea*	CdCl_2_	Seedlings	50 mg/L, 300 mg/L and 500 mg/L	100 µM	Improved the plant growth by mitigation of Cd induced oxidative stress	^126^
*Lupinus luteus*	CdCl_2_	Roots	50 µM −100 µM	10 µM	Reduced the Cd toxicity and improved the level of antioxidants	^71^
*Oryza sativa*	CdCl_2_	Seedlings	10 µM	30 µM	Enhanced the plant biomass and upregulation of ROS scavenging enzyme and maintained the cellular ionic homeostasis	^127^
*Typha angustifolia*	CdCl_2_	Seedlings	445 µM	100 µM	Reduced the Cd induced toxicity and enhanced accumulation of antioxidants	^128^
*Arabidopsis thaliana*	CuSO_4_	Plants	5 µM, 25 µM or 50 µM	10 µM	Increased the viability of cells	^129^
*Oryza sativa*	CuSO_4_	Seedlings	100 µM	200 µM	Enhanced the activity of ROS quenchers	^130^
*Triticum aestivum*	Ni Cl_2_	Seedlings	100 µM	200 µM	Inhibition of oxidative burst by the upregulation of antioxidants	^27^)
*Lolium perenne*	Pb(NO_3_)_2_	Seedlings	500 µM	50 µM, 100 µM and 200 µM	Improved the photosynthetic pigment and ROS scavengers level	^131^
*Triticum aestivum*	Pb(NO_3_)_2_	Roots	50 µM and 250 µM	100 µM	Raised the accumulation pattern of antioxidants	^132^
*Triticum aestivum and Phaseolus vulgaris*	ZnSO_4_	Seedling	0–21.6 µM	100 µM	Reduced the Cd induced toxicity by decreasing the MDA and H_2_O_2_ contents and enhanced the activity of antioxidant	^113^

In rice seedlings, the alleviation of cadmium toxicity induced by calcium was shown to be mediated by endogenous nitric oxide mediates.^138^ In *A. thaliana*, Cd-induced flowering was delayed by SNP, and this was directly related to the augmented level of NO in leaves tissue.^139^ According to^140;141,118^ the application of SNP can induce ROS-regulated Cd toxicity in *B. juncea*. Pre-treatment of NO triggers the rapid seed germination and seedling growth of *O. sativa* upon Cd exposure.^142^ Cd-induced oxidative stress can reduce *S*-nitrosylation of CAT, which ultimately enhances the CAT activity and ROS detoxification. Thus, S-nitrosylation can modulate the accumulation of ROS by regulating antioxidant defense metabolism and reactive oxygen species generating enzymes.^143^

Exogenously applied NO minimizes the Cu-induced phytotoxicity by detoxification of ROS in seedlings of *Oryza sativa*, and this can be repressed by NO scavenger cPTIO.^144^ In addition, in Cu-treated *T. aestivum*, the germination of their seeds and the activity of antioxidant enzymes such as SOD and CAT were improved by using NO donor.^145^ In the roots of *Vicia faba*, Cu toxicity was efficiently prevented also by the application of SNP.^111^ The antioxidant and detoxification properties of NO were shown in Cd^2+^ and Cu^2+^ treated suspension l culture of *Glycine max*.^146^ Singh et al. and Ismail.^114,120^ found that the ameliorative effect of NO against arsenic-induced cytotoxicity in *O. sativa* and *V. radiata* is caused by the reduction of RODS and MDA levels which activate antioxidant defenses. The study by Kazemi et al.^112^ on *Brassica napus* leaves showed that the exogenous NO in combination with salicylic acid increased the photosynthetic pigment content, enhanced the activities of antioxidants, reduced the level of hydrogen peroxide, malonaldehyde and caused osmolyte accumulation under Ni toxicity. In summary, the antioxidant molecule NO counteracts ROS by improving the activity of antioxidant enzymes.

## Temperature stress

Temperature stress severely affects the distribution and survival of plant species worldwide. There are numerous reports demonstrating detrimental effect of extremely high and low temperatures on plants at the physiological and biochemical, and molecular levels. In general, plants exposed to temperature stress show enhanced accumulation of ROS, leading to oxidative damage (Figure 4). Several studies reported that plants also acclimate to high-temperature stress by interacting ROS with NRS. Plants exposed to low concentrations of NO have improved their heat stress tolerance.^147,148^

NO production was induced during the heat stress, and this response is essential for plant tolerance to heat stress. Application of NO suppressed high temperature-induced symptoms in *Oryza sativa* and increased the acclimation rate of *Triticum aestivum* and *Zea mays*.^62,74^ The antioxidant properties of NO are demonstrated by its ability to reduce ROS levels and trigger activities of antioxidants, including superoxide dismutase, catalase and ascorbate peroxidase under extremely high temperatures.^149^ Similarly, the induction of heat shock gene expression has been reported to attenuate ROS accumulation and is also involved in establishing cellular homeostasis under high-temperature stress.^61^ On the other hand, *HOTS/ATGSNOR* proteins identified in plants which are sensitive to heat and increased the level of nitrate and *S*-nitrosothiols. Previous studies confirmed that *hot5/atgsnor1* and (*nox1/cue1*) (NO overproducing lines) are heat-sensitive protein which involved in the thermo tolerance mechanism by regulation of NO/SNO.^150,151^

In *Pisum sativum* and *Brassica juncea*, the heat stress stimulated the metabolism of RNS and increased the SNO content.^45^ The study by Ziogas et al.^98^ in citrus leaves demonstrated the significant increase in NO, SNO and superoxide levels to be directly associated with a decrease in chlorophyll pigment contents and enhanced electrolyte leakage. This report suggests that NO/RNS homeostasis is required for acclimation to heat stress. The NO/RNS homeostasis can be disturbed by the accumulation of ROS/RNS, which leads to oxidative and nitrosative damage under heat stress.

## Conclusion and perspectives

The advancement of research emphasizing on NO biology in plants exhibits a plethora of biological functions including plant growth, development and response to different abiotic stresses. Thus, alteration in redox homeostasis was interrupted by every aspects of plant biology. While there has been considerable progress in enlightening the role of NO in different abiotic stresses, many challenges remain unclear. Future investigation on NO biology should emphasize on the cross talk between various developmental and defense hormones and also explored the nitric oxide functions in abiotic stress. Additionally, endogenous levels of NO influence nutrient such as nitrogen concentration which is essential macroelement in plant development. Hence, some future attention permits that the understanding behind the biosynthesis, assimilation, and NO turnover might be interconnected.

Moreover, nitric oxide signaling is transient in nature. In the context of NO bioactivity, GSNOR enzyme indirectly regulates the level of *S*-nitrosylation by turning over *S*-nitrosoglutathione and hence its activity apparently reduces the NO signaling specificity. In conclusion, copious studies have disclosed exciting innovative area of investigation for the plant researchers working on cellular integration. NO acts as key retrograde signals and antioxidants among various compartments of plant cell and also modulates gene expression which assists plant cell acclimation to environmental fluctuation. Future research should be focus to unravel the mystery of functional aspects of NO in plant cell. The study of free redox radical, i.e., NO, is extremely important for plant biologists as more attention is required to get an insight into underlying redox-based molecular machinery to discover that NO possesses a role in regulation of plant cell defense against different abiotic stresses.

## Abbreviations

NO: Nitric oxide
SNP: Sodium nitroprusside
SNAP: S-nitroso-N-acetylpenicillamine
DETA: 2,2′(hydroxyl nitroso hydrazono) bis-ethanimine
cPTIO: 2-(4-Carboxyphenyl) − 4, 4, 5, 5 tetramethylimidazoline-1-oxyl-3-oxide
NOS: Nitric oxide synthase
NR: Nitrate reductase
NiR: Nitrite reductsae
Ni:NOR: Nitrite nitric oxide reductase
XOR: Xanthine oxidoreductase
XOD: Xanthine oxidase
XDH: Xanthine dehydrogenase
SNO: *S*-nitrosothiol
GSNO: *S*-nitrosoglutathione
GSNOR: *S*-nitrosoglutathione reductase
GSH: Glutathione
SOD: Superoxide dismutase
CAT: Catalase
APX: Ascorbate peroxidase
GPX: Guaiacol peroxidase
H_2_O_2_: Hydrogen peroxide
O_2_: Superoxide radical
OH: Hydroxyl radical
ONOO: Peroxynitrite
ROS: Reactive oxygen species
NADH: Nicotinamide adenine dinucleotide hydride
NaHCO_3_: Sodium bicarbonate
KNO_3_: Potassium nitrate
CaSO_4_: Calcium sulphate
MgSO_4_: Magnesium sulphate
SnRK2.6: Sucrose non fermenting related protein kinase
RBOH: Respiratory burst oxidase homolog
Cys: Cysteine
PC: Phytochelatin
ABA: Abscisic acid
